# Prevalence of low back pain and work-related musculoskeletal disorders in Brazilian domestics and cleaners: 2013 and 2019 National Health Survey

**DOI:** 10.47626/1679-4435-2021-887

**Published:** 2023-08-08

**Authors:** Rodrigo Suguimoto Iwami, Marcus Tolentino Silva, Cristiane de Cássia Bergamaschi

**Affiliations:** 1 Programa de Pós-Graduação em Ciências Farmacêuticas, Universidade de Sorocaba, Sorocaba, SP, Brasil

**Keywords:** low back pain, cumulative trauma disorders, housekeeping, epidemiology, dor lombar, transtornos traumáticos cumulativos, zeladoria, epidemiologia

## Abstract

**Introduction:**

Low back pain and work-related musculoskeletal disorders are two of the leading causes of absenteeism worldwide.

**Objectives:**

To determine the prevalence and predictive factors of low back pain and work-related musculoskeletal disorders in Brazilian domestics and cleaners.

**Methods:**

This population-based cross-sectional study used data from the 2013 and 2019 National Health Survey (Pesquisa Nacional de Saúde), involving a total of 8,160 workers. The prevalence of low back pain and work-related musculoskeletal disorders was calculated based on adjusted prevalence ratio by Poisson regression and 95%CI.

**Results:**

The prevalence of lowback pain was 19.1% in 2013 and 20.6% in 2019, while the prevalence of musculoskeletal disorders was 2.2% in 2013 and 2.4% in 2019. Low back pain was associated with older age (prevalence ratio: 1.74; 95%CI 1.44-2.09), poor or very poor self-rated health (prevalence ratio: 2.10; 95%CI 1.76-2.50), holding a prepaid health plan (prevalence ratio: 1.27; 95%CI 1.09-1.47), and moderately severe (prevalence ratio: 2.27; 95%CI 1.84-2.80) or severe (prevalence ratio: 2.32; 95%CI 1.77-3.04) depressive symptoms. Musculoskeletal disorders affected domestics less frequently (prevalence ratio: 0.53; 95%CI 0.40-0.72) and were associated with women (prevalence ratio: 2.50; 95%CI 1.34-4.66), adults (40-59 years) (prevalence ratio: 1.79; 95%CI 1.26-2.55), holding a prepaid health plan (prevalence ratio: 2.31; 95%CI 1.63-3.26), and the presence of moderately severe (prevalence ratio: 4.00; 95%CI 2.34-6.86) or severe (prevalence ratio: 3.63; 95%CI 1.77-7.46) depressive symptoms.

**Conclusions:**

Brazilian domestics and cleaners need interventions and improvements in health care given the prevalence of low back pain and musculoskeletal disorders as well as their association with depression.

## Abstract (pt)

**Introdução:**

Lombalgia e distúrbios osteomusculares relacionadas ao trabalho são duas das principais causas de absenteísmo no mundo.

**Objetivos:**

Determinar a prevalência e fatores preditivos da lombalgia e distúrbios osteomusculares relacionados ao trabalho em trabalhadores domésticos e faxineiros brasileiros.

**Métodos:**

Este estudo transversal populacional coletou dados da Pesquisa Nacional de Saúde, realizada nos anos de 2013 e 2019, totalizando uma amostra de 8.160 trabalhadores. A prevalência da lombalgia e distúrbios osteomusculares relacionados ao trabalho autorreferidos foi calculada pela razão de prevalência ajustada pelo modelo de regressão de Poisson e IC95%.

**Resultados:**

A prevalência de lombalgia foi de 19,1% em 2013 e 20,6% em 2019; e de distúrbios osteomusculares foi de 2,2% em 2013 e 2,4% em 2019. A lombalgia esteve associada aos indivíduos idosos (razão de prevalência: 1,74; IC95% 1,44-2,09), com percepção baixa ou muito baixa a respeito da sua saúde (razão de prevalência: 2,10; IC95% 1,76-2,50), com plano de saúde (razão de prevalência: 1,27; IC95% 1,09-1,47) e com sintomas depressivos moderadamente graves (razão de prevalência: 2,27; IC95% 1,84-2,80) ou graves (razão de prevalência: 2,32; IC95% 1,77-3,04). A presença de distúrbios osteomusculares foi menor nos trabalhadores domésticos (razão de prevalência: 0,53; IC95% 0,40-0,72); e esteve associada ao sexo feminino (razão de prevalência: 2,50; IC95% 1,34-4,66), adultos (40-59 anos) (razão de prevalência: 1,79; IC95% 1,26-2,55), ter plano de saúde (razão de prevalência: 2,31; IC95% 1,63-3,26) e presença de sintomas depressivos moderadamente graves (razão de prevalência: 4,00; IC95% 2,34-6,86) ou graves (razão de prevalência: 3,63; IC95% 1,77-7,46).

**Conclusões:**

Trabalhadores domésticos e faxineiros necessitam de intervenções, melhorias na saúde e adaptação dos ambientes de trabalho dada a prevalência de lombalgia e distúrbios osteomusculares e associações com a depressão.

## INTRODUCTION

Low back pain may result from different musculoskeletal diseases, intervertebral disc disorders, spondylitis, or radiculopathies.^1^ Despite its high burden globally there is a lack of attention and research dedicated to low back pain in developing countries compared with developed nations.^2,3^ Information from the Brazilian Social Security System shows high rates of disability retirement due to back pain in Brazil.^1^

Work-related musculoskeletal disorders rank among the main chronic non-communicable diseases in Brazil. They can be caused by excessive use of part of the musculoskeletal system or by occupation-related physical activities, representing a high cost to the public sector.^4^ These disorders encompass a large number of inflammatory and degenerative diseases, including myalgia, tendinitis, tenosynovitis, and degenerative musculoskeletal disorders.^5^

Domestic workers can be defined as individuals who provide a person or family, at their residence, with continuous, paid or unpaid personal services for more than two days per week.^6^ Although domestics and cleaners share similarities in their occupational profile, such as spending considerable time standing, walking or kneeling, quick movements of the upper extremities, repetitive activities, and movements while holding tools and equipment, they differ from each other. Housework can be less repetitive but takes longer to complete, resulting in higher exposure to risk factors and less opportunity to recover.^7^

Information about low back pain and work-related musculoskeletal disorders in the Brazilian population has been provided by the National Health Survey (Pesquisa Nacional de Saúde [PNS]). The latest surveys were conducted by the Ministry of Health in 2013 and 2019 and provided a description of Brazilian self-rated health and lifestyles, including access and utilization of health care services, preventive care, continuity of care, and health care funding.^8,9^ The questionnaire consists of three phases of data collection: concerning the household; the residents; and an interview with an adult resident about self-rated health.^9,10^

A cross-sectional study analyzing data from the 2013 PNS showed that 18.5% of the Brazilian population had chronic back pain.^1^ Another cross-sectional study reported that 2.4% of Brazilian adults suffered from work-related musculoskeletal disorders. This population consisted mainly of adult women (aged 30-59 years) and urban dwellers.^11^ Another Brazilian study identified the predictive factors of work-related musculoskeletal disorders as being a woman, temporarily away from work, and exposed to noise at the workplace, holding the same job for at least 4.5 years, engaging in volunteer work, having a diagnosis of arthritis or rheumatism, and suffering from depression.^12^

No epidemiological studies investigating the association of these diseases in Brazilian domestics and cleaners are available. Therefore, the present study aimed to determine the prevalence and predictive factors of low back pain and work-related musculoskeletal disorders in Brazilian domestics and cleaners, based on data collected in the 2013 and 2019 PNS.

## METHODS

### STUDY DESIGN

This population-based cross-sectional study analyzed data from the PNS database, a representative, nationwide, household survey that collected data from the Brazilian population in 2013 and 2019. The outcomes of interest were low back pain and work-related musculoskeletal disorders in the population of domestics and cleaners.

### RESEARCH SITE AND SETTING

The PNS was conducted by the Brazilian Institute of Geography and Statistics (Instituto Brasileiro de Geografia e Estatística [IBGE]) in collaboration with the Ministry of Health in 2013 and 2019.^8,9^ The household samples were drawn from the master sample of the National Household Sample Survey (Pesquisa Nacional por Amostra de Domicílio [PNAD]), an annual survey used by the IBGE for planning Federal government actions. This approach provided broader geographic coverage and improved the accuracy of estimates.^13^ Details of the PNS methods have been described elsewhere.^8,9,14^

### SAMPLE SELECTION

There was approximately 201 million people living in Brazil in 2013^15^ and 210 million in 2019,^16^ and the total sample size was 81,357 households in 2013 and 84,073 in 2019.

Sample selection in the PNS was performed in three stages: census tracts, households, and residents aged ≥ 18 years (in 2013)^13^ and ≥ 15 years in 2019.^9^ Sectors with special characteristics and low population density were excluded (indigenous communities, barracks, military bases, lodgings, camps, boats, penitentiaries, penal colonies, prisons, nursing homes, orphanages, convents, and hospitals).^17^ The present study selected interviews containing responses to questions about low back pain and work-related musculoskeletal disorders involving domestics and cleaners.

### DATA COLLECTION PROCEDURE

The questionnaires were completed by interviewers previously trained by the IBGE and Ministry of Health staff with handheld computers. The results are available at the IBGE website (http://www.ibge.gov.br/home/estatistica/populacao/pns/2013_vol3/default_microdados.shtm).

The prevalence of low back pain and work-related musculoskeletal disorders was self-reported and derived from the responses to the following questions: “Do you have any chronic back issues, such as chronic back or neck pain, low back pain, sciatica, and vertebral or intervertebral disc disorders?” and “Has a doctor ever diagnosed you with a work-related musculoskeletal disorder?” The possible answers were “Yes” or “No.”

### PREDICTIVE FACTORS OF LOW BACK PAIN AND WORK-RELATED MUSCULOSKELETAL DISORDERS

Relevant information was extracted from the data provided by the PNS to determine the predictive factors of self-reported low back pain and work-related musculoskeletal disorders (dependent variables).

The chosen independent variables were: (1) sociodemographic: sex (male or female); age (18-39, 40-59, or ≥ 60 years); ethnicity (black/brown/indigenous or white/Asian); marital status (married, single, or separated/divorced/widowed); education (university/high school completed, primary school completed, or no education/incomplete primary school); and occupation (domestic or cleaner); (2) clinical: self-rated health (very good/good, regular, or poor/very poor); holding a prepaid health plan; and presence of depressive symptoms.^18^

The Patient Health Questionnaire-9 (PHQ-9) was used to measure depressive symptoms in the Brazilian population.^19^ The questionnaire consists of nine questions, each scored as 0 (not at all), 1 (several days), 2 (more than half the days), or 3 (nearly every day). Depending on the final PHQ-9 score, the respondents were stratified by symptom severity into none-minimal (0-3 points), mild (5-9 points), moderate (10-14 points), moderately severe (15-19 points), and severe (20-27 points).

Questions from the PNS that refer to the PHQ-9 questionnaire are as follows: (1) Over the last 2 weeks, how often have you had trouble sleeping, such as difficulty falling asleep, frequent night awakenings, or sleeping more than usual?; (2) Over the last 2 weeks, how often have you had problems due to not feeling rested or lacking vitality throughout the day, such as feeling tired and having little energy?; (3) Over the last 2 weeks, how often have you shown little interest or pleasure in doing things?; (4) Over the last 2 weeks, how often have you had trouble focusing on your regular activities?; (5) Over the last 2 weeks, how often have you had trouble eating, such as having poor appetite or eating a lot more than usual?; (6) Over the last 2 weeks, how often have you experienced speaking or moving slowly, or being very restless or fidgety?; (7) Over the last 2 weeks, how often have you felt depressed, “down,” or hopeless?; (8) Over the last 2 weeks, how often have you felt bad about yourself, feeling that you are a failure or thinking that you have let your family down?; and (9) Over the last 2 weeks, how often have you thought about hurting yourself in some way or that you would be better off dead?

### STATISTICAL ANALYSIS

The complex sampling design was used in all analyses. The characteristics of the participants were first described using balanced frequencies and stratified by the presence of low back pain and work-related musculoskeletal disorders. To identify the predictive factors of both diseases, the prevalence ratio (PR) for a 95%CI was calculated using Poisson regression with robust variance. The PR was adjusted if the variables presented p < 0.05 (significance level of 5%). Associations were considered significant when p < 0.05. All calculations were performed using the STATA software (version 14.2) package.

### ETHICAL ASPECTS

The PNS was approved by the National Ethics Commission (Comissão Nacional de Ética em Pesquisa [CONEP]) (# 328,159) on June 26, 2013, and (# 3,529,376) on August 23, 2019. All the participants were volunteers, and data confidentiality was assured.^9,20^

## RESULTS

Of the study sample of 3,511 people in 2013 and 4,649 in 2019, 67.3% (2013) and 69.5% (2019) were domestics and 32.7% (2013) and 30.5% (2019) were cleaners, accounting for 5.8% of 60,202 interviewees in 2013 and 5.1% of 90,846 interviewees in 2019.

The prevalence of low back pain was 19.1% in 2013 and 20.6% in 2019. The prevalence of work-related musculoskeletal disorders was 2.2% in 2013 and 2.4% in 2019.

The sample characteristics are presented in Tables 1 and 2. In both years, the population consisted mainly of adult women of black, brown, or indigenous ethnicity, single, with no education or incomplete primary school, very good or good self-rated health, holding no prepaid health plan and presenting minimal depressive symptoms.

**Table 1:** Sample characteristics of the Brazilian population of cleaners and domestics (n = 3,511 participants) in 2013

Variables	Population n(%)	LBP n(%)	p-value	WMSD n(%)	p-value
Occupation					
Cleaner	1149 (32.7)	217 (18.9)	0.813	34 (3.0)	0.031
Domestic	2,362 (67.3)	454 (19.2)		43 (1.8)	
Sex					
Male	473 (13.5)	76 (16.1)	0.07	1 (0.2)	0.002
Female	3,038 (86.5)	595 (19.6)		76 (2.5)	
Age (years)					
18-39	1,742 (49.6)	259 (14.9)	< 0.001	21 (1.2)	< 0.001
40-59	1,550 (44.1)	359 (23.2)		54 (2.5)	
>60	219 (6.2)	53 (24.2)		2 (0.9)	
Ethnicity					
White/Asian	1,036 (29.5)	210 (20.3)	0.258	27 (2.6)	0.280
Black/brown/indigenous	2,475 (70.5)	461 (18.6)		50 (2.0)	
Marital status					
Married	1,051 (29.9)	228 (21.7)	0.002	32 (3.0)	< 0.001
Separated/divorced/widowed	518 (14.7)	112 (21.6)		19 (3.7)	
Single	1,942 (55.3)	331 (17.0)		26 (1.3)	
Education					
High school completed/university	878 (25.0)	140 (15.9)	0.008	15 (1.7)	0.525
Primary school completed	775 (22.1)	143 (18.4)		18 (2.3)	
No education/incomplete primary school	1,858 (52.9)	388 (20.9)		44 (2.4)	
Self-rated health					
Very good/good	2,122 (60.4)	274 (12.9)	< 0.001	31 (1.5)	0.001
Regular	1,193 (34.0)	312 (26.1)		40 (3.3)	
Poor/very poor	196 (5.6)	85 (43.4)		6 (3.1)	
Prepaid health plan					
No	3,098 (88.2)	574 (18.5)	0.016	54 (1.7)	< 0.001
Yes	413 (11.8)	97 (23.5)		23 (5.6)	
Depressive symptoms					
Minimal	2,533 (72.1)	351 (13.9)	< 0.001	36 (1.4)	< 0.001
Mild	592 (16.9)	161 (27.2)		23 (3.9)	
Moderate	252 (7.2)	106 (42.1)		14 (5.6)	
Moderately severe	83 (2.4)	32 (38.5)		2 (2.4)	
Severe	51 (1.4)	21 (41.2)		2 (3.9)	

LBP = low back pain; WMSD = work-related musculoskeletal disorders.

**Table 2:** Sample characteristics of the Brazilian population of cleaners and domestics (n = 4,649 participants) in 2019

Variables	Population n (%)	LBP n (%)	p-value	WMSD n (%)	p-value
Occupation					
Cleaner	1,420 (30.5)	286 (20.1)	0.586	53 (3.7)	< 0.001
Domestic	3,229 (69.5)	673 (20.8)		60 (1.9)	
Sex					
Male	796 (17.1)	130 (16.3)	0.001	10 (1.3)	0.018
Female	3,853 (82.9)	829 (21.5)		103 (2.7)	
Age (years)					
18-39	1,753 (37.7)	242 (13.8)	< 0.001	28 (1.6)	0.007
40-59	2,464 (53.0)	585 (23.7)		76 (3.1)	
>60	432 (9.3)	132 (30.6)		9 (2.1)	
Ethnicity					
White/Asian	1,244 (26.8)	266 (21.4)	0.442	41 (3.3)	0.021
Black/brown/indigenous	3,405 (73.2)	693 (20.3)		72 (2.1)	
Marital status					
Married	1,351 (20.1)	293 (21.7)	< 0.001	39 (2.9)	0.294
Separated/divorced/widowed	746 (16.0)	187 (25.1)		20 (2.7)	
Single	2,552 (54.9)	479 (18.8)		54 (2.1)	
Education					
High school completed/university	1,422 (30.6)	241 (16.9)	< 0.001	32 (2.2)	0.602
Primary school completed	940 (20.2)	173 (18.4)		27 (2.9)	
No education/incomplete primary school	2,287 (49.2)	545 (23.8)		54 (2.4)	
Self-rated health					
Very good/good	2,805 (60.3)	378 (13.5)	< 0.001	48 (1.7)	< 0.001
Regular	1,616 (34.8)	478 (29.6)		49 (3.0)	
Poor/very poor	228 (4.9)	103 (45.2)		16 (7.0)	
Prepaid health plan					
No	4,220 (90.8)	845 (20.0)	0.001	88 (2.1)	< 0.001
Yes	429 (9.2)	114 (26.6)		25 (5.8)	
Depressive symptoms					
Minimal	3,192 (68.7)	475 (14.9)	< 0.001	43 (1.3)	< 0.001
Mild	835 (18.0)	237 (28.4)		31 (3.7)	
Moderate	373 (8.0)	134 (35.9)		16 (4.3)	
Moderately severe	169 (3.6)	74 (43.8)		16 (9.5)	
Severe	80 (1.7)	39 (48.7)		7 (8.7)	

LBP = low back pain; WMSD = work-related musculoskeletal disorders.

In both years, the population with low back pain consisted predominantly of older adults, married or separated/divorced/widowed, with no education/incomplete primary school, poor or very poor self-rated health, and without prepaid health plan (p ≤ 0.05). The presence of moderate-to-severe depressive symptoms was significant in this population (p < 0.001). There was no significant difference in the number of men and women in 2013, but more women were interviewed in 2019.

In the population with work-related musculoskeletal disorders, in both years, most interviewees were separated/divorced/widowed, with poor or very poor self-rated health and moderate-to-severe depressive symptoms (p < 0.001). Regarding age, the sample consisted mainly of adults in 2013 and older adults in 2019.

The regression analysis was performed using both 2013 and 2019 data from Brazilian domestics and cleaners (Table 3).

**Table 3:** Predictive factors of low back pain (n = 1,630 participants) and work-related musculoskeletal disorders (n = 190 participants) in domestics and cleaners (Brazil, 2013 and 2019)

Variables	LBP Adjusted PR (95%CI)	p-value	WMSD Adjusted PR (95%CI)	p-value
Year				
2013	1.00	0.771	1.00	0.572
2019	1.02 (0.92-1.12)		1.09 (0.81-1.46)	
Occupation				
Cleaner	1.00	0.555	1.00	< 0.001
Domestic	0.97 (0.87-1.08)		0.53 (0.40-0.72)	
Sex				
Male	1.00	0.601	1.00	0.004
Female	1.04 (0.89-1.21)		2.50 (1.34-4.66)	
Age (years)				
18-39	1.00	< 0.001	1.00	0.002
40-59	1.39 (1.24-1.56)		1.79 (1.26-2.55)	
>60	1.74 (1.44-2.09)		1.07 (0.54-2.14)	
Ethnicity				
White/Asian	1.00	0.777	1.00	0.088
Black/brown/indigenous	0.98 (0.88-1.10)		0.77 (0.57-1.04)	
Marital status				
Married	1.00	0.103	1.00	0.143
Separated/divorced/widowed	0.95 (082-1.10)		0.96 (0.65-1.43)	
Single	0.89 (0.79-099)		0.73 (0.52-1.02)	
Education				
High school completed/university	1.00	0.177	1.00	0.309
Primary school completed	1.08 (0.93-1.25)		1.38 (0.91-2.08)	
No education/incomplete primary school	1.13 (099-1.28)		1.19 (0.83-1.71)	
Self-rated health				
Very good/good	1.00	< 0.001	1.00	0.143
Regular	1.68 (1.51-1.88)		1.42 (1.03-1.97)	
Poor/very poor	2.10(1.76-2.50)		1.63 (0.97-2.74)	
Prepaid health plan				
No	1.00	0.002	1.00	< 0.001
Yes	1.27 (1.09-1.47)		2.31 (1.63-3.26)	
Depressive symptoms				
Minimal	1.00	< 0.001	1.00	< 0.001
Mild	1.69 (1.49-1.91)		2.40 (1.68-3.42)	
Moderate	2.12 (1.82-2.47)		2.60 (1.67-4.07)	
Moderately severe	2.27 (1.84-2.80)		4.00 (2.34-6.86)	
Severe	2.32 (1.77-3.04)		3.63 (1.77-746)	

LBP = low back pain; PR = prevalence ratio; WMSD = work-related musculoskeletal disorders.

The presence of low back pain in this population (n = 671 participants in 2013 and n = 959 in 2019) was associated with older age, poor or very poor self-rated health, holding a prepaid health plan, and having moderately severe or severe depressive symptoms.

Furthermore, being a cleaner, woman, aged 40-59 years, holding a prepaid health plan and showing moderately severe or severe depressive symptoms was associated with the presence of work-related musculoskeletal disorders in the population of Brazilian domestics and cleaners (n = 77 participants in 2013 and n= 113 in 2019).

## DISCUSSION

### SUMMARY OF FINDINGS AND INTERPRETATION BASED ON AVAILABLE LITERATURE

The information generated by this study considered data from the Brazilian population of domestics and cleaners from the latest two PNS conducted in 2013 and 2019. This population consisted mainly of adult women of black/brown/indigenous ethnicity, single, with no education or incomplete primary school, very good or good self-rated health, holding no prepaid health plan and having minimal depressive symptoms.

The prevalence of low back pain (19.1% in 2013 and 20.6% in 2019) was higher than that of work-related musculoskeletal disorders (2.2% in 2013 and 2.4% in 2019). Low back pain was associated with older adults with poor or very poor self-rated health, holding a prepaid health plan and having moderately severe or severe depressive symptoms. Work-related musculoskeletal disorders were associated with being a cleaner, woman, aged 40 to 59 years, holding a prepaid health plan and having moderately severe or severe depressive symptoms.

A study also analyzing data from the 2013 PNS showed that approximately one-fifth of the Brazilian population reported chronic back pain, where this condition was more frequent in women than in men.^1^ Another study analyzing the 2013 PNS reported a higher prevalence of low back pain after the age of 60, and it was associated with the Brazilian population having a low level of education or no schooling.^21^ Similar results were observed for the population analyzed in the present study.

A cross-sectional study conducted between 2011 and 2013 at the Workers’ Health Reference Center (Centro de Referência em Saúde do Trabalhador [CEREST]) in Guarulhos (São Paulo state), with a population of 192 workers with work-related musculoskeletal disorders and occupational low back pain, showed that most participants performed cleaning activities as cleaning assistants, domestics, or cleaners (15.6%), followed by construction workers (13%).^22^ This result highlights the need for studies that involve the population of domestics and cleaners, given the high prevalence rates of both of these diseases, especially low back pain.

A population-based survey conducted by IBGE, known as “Family and gender roles” (result of Brazils affiliation to the International Social Survey Program [ISSP]), characterized Brazilian domestics in 2011. Out of a total 6,516,996 participants, most were women (92%), black (56.6%), and had a low level of education (20.5% had not completed primary school and 35.6% had < 1 year of schooling)^23^; similar results were observed in the present study population.

A study involving 11,660 housekeepers from the United States hotel industry, conducted between 2003 and 2005, found that this population had the highest rates of injury and musculoskeletal disorders among hotel workers; this population also consisted mainly of women (97.7%) and black individuals (29.5%).^24^

A study also based on data from the 2013 PNS reported a prevalence of 2.5% of work-related musculoskeletal disorders in the Brazilian population. Risk factors were being a woman, temporarily away from work, and exposed to noise at the workplace, holding the same job for at least 4.5 years, engaging in volunteer work, having a diagnosis of arthritis or rheumatism, and suffering from depression.^12^ Although the study populations differ, the present study identified that being a woman and having depressive symptoms were also risk factors for work-related musculoskeletal disorders in Brazilian domestics and cleaners.

A cross-sectional study of 2,231 adults from Kayseri (in Turkey) investigated risk factors for low back pain and their association with depression. Domestics were the second group most affected by low back pain. Furthermore, being an adult, woman, smoker, and from low socioeconomic status, having depression and living in a rural area were factors associated with low back pain.^25^

In the present study, the presence of moderate or severe depressive symptoms was associated with low back pain.

Another cross-sectional study determined the prevalence of pain in 941 hotel room cleaners in Las Vegas, United States. Severe or very severe low back pain was the most prevalent pain (63%), followed by upper back pain (59%). The study found that older age was associated with lower levels of low back pain,^26^ contradicting the findings of the present study. The study also found that handling heavy equipment, inadequate cleaning supplies, and lack of squeegees and mops were risk factors for low back pain.^26^

Another cross-sectional study used a database to identify incidents of occupational injury among cleaners in the health care sector. The study was conducted between 2004 and 2005 and found a total of 145 injuries, 86 of which (59%) were musculoskeletal. Similarly, most of the injuries involved women (90%), adults aged 40-49 years (47%), full-time employees (47%), people who worked in acute care (86%), and those who had 1-5 years of experience (35%). Being a woman, being an inexperienced worker and working in acute care were the greatest risk factors for all injuries.^27^

The scarcity of national studies on the specific population of domestics and cleaners underscores the relevance of the data gleaned by the present study. Moreover, few international studies were found associating these diseases and predictive factors in this population. The finding that both diseases were associated with individuals holding a prepaid health plan can be explained by their greater access to diagnostic and health care services. The finding that musculoskeletal disorders were more commonly present in women than in men can be explained by the fact that women spend more time than men in domestic activities and are therefore more likely to develop these diseases. In addition, frequent lifting of heavy objects and psychosocial stress tend to exacerbate these disorders.^7^

### STRENGTHS AND LIMITATIONS

A higher prevalence of low back pain than of work-related musculoskeletal disorders was observed in the Brazilian population of domestics and cleaners. However, it is important to highlight that these results may be biased, since cross-sectional studies based on household interviews might not reflect the entire population of a specific area, as the people interviewed were those present at the time of data collection. Also, the fact that the diagnosis was self-reported or unrecognized may lead to lower prevalence rates by this method. However, the large size of the survey rendered it impractical to use a two-step data collection process for diagnostic confirmation.

Cross-sectional studies play an important role in collecting data from the population and can greatly aid the development of new health care services and future studies that can infer causality from the findings. The scarcity of electronic databases providing this type of information and the fact that the present study collected data for two occupations in great need of health interventions underscores the relevance of the findings.

The PNS is a highly representative nationwide household survey. The household samples, drawn from the master sample of the PNAD stored in the Household Survey Integrated System (Sistema Integrado de Pesquisas Domiciliares [SIPD]) of the IBGE, provided broader geographic coverage and improved the accuracy of estimates.^13^ In addition, the use of a validated instrument to assess depressive symptoms (PHQ-9) demonstrates the care taken in devising the questionnaire.

### IMPLICATIONS FOR CLINICAL PRACTICE AND RESEARCH

Low back pain is a common health problem that generates high costs worldwide,^3^ is the second most reported disease in the Brazilian population,^28^ and is responsible for high rates of disability retirement in Brazil.^29^ In the present study, low back pain was more prevalent than work-related musculoskeletal disorders in the population assessed.

Low back pain and work-related musculoskeletal disorders are conditions that frequently affect Brazilian domestics and cleaners, hence the need for studies investigating the risk factors associated with both diseases in this population. Thus, interventions to prevent low back pain in this population should prioritize older adults, individuals with poor or very poor self-rated health, and those with depressive symptoms. For work-related musculoskeletal disorders, preventive actions should focus on women, aged 40 to 59 years, who hold a prepaid health plan and have depressive symptoms.

This information is important to guide future public health policies to prioritize more vulnerable groups and to assist health care services in planning preventive actions and treatment strategies for the Brazilian population, thereby improving workers’ quality of life and reducing absenteeism and its associated cost.

## CONCLUSIONS

These findings underscore the need for health interventions, both physical and psychological, targeting Brazilian domestics and cleaners given the prevalence of low back pain and work-related musculoskeletal disorders in this population, as well as their association with depression. Future public health policies should prioritize these vulnerable groups.
